# Clinical Characteristics of Distinct Subgroups of Patients with Primary Sjögren’s Syndrome Classified by Serological Profiles: A Comparison Study

**DOI:** 10.3390/jpm14090967

**Published:** 2024-09-12

**Authors:** Erdal Bodakçi

**Affiliations:** Division of Rheumatology, Department of Internal Medicine, Eskisehir Cıty Hospıtal, Eskisehir 26100, Turkey; drebodakci@gmail.com; Tel.: +90-222611-40-00-21063

**Keywords:** primary Sjögren’s syndrome, autoantibodies, clinical symptoms

## Abstract

Sjögren’s syndrome (SS) is an autoimmune disease characterized by heterogeneous clinical presentation and the presence of various autoantibodies. This study aimed to determine the differences in clinical findings according to antibody positivity in patients with primary Sjögren syndrome (pSS) in the Turkish population. A retrospective study was conducted and 402 patients (378 women and 24 men) with pSS were analyzed. The patients were categorized into three subgroups based on serological tests. These were (1) quadruple seropositivity (positive for anti-Sjögren’s syndrome-related antigen A antibodies (anti-SSA; anti-Ro) and anti-Sjögren’s syndrome-related antigen B antibodies (anti-SSB; anti-La), rheumatoid factor (RF), and antinuclear antibody (ANA); (2) double seropositivity (positive for ANA and anti-SSA/Ro antibodies); and (3) quadruple seronegativity (negative for ANA, RF, anti-SSA/Ro and anti-SSB/La antibodies). The number of quadruple-seropositive patients was 72 (18.6%), double-seropositive 174 (43.2%), and quadruple-seronegative was 85 (21.1%). The age at diagnosis of quadruple-seropositive pSS was 42.4 ± 10.8, which was significantly younger than that of patients with double-seropositive and quadruple-seronegative pSS (*p* = 0.021, *p* = 0.112). In terms of organ involvement, salivary gland enlargement, arthralgia, arthritis, Raynaud’s phenomenon, lymphadenopathy, cutaneous vasculitis, interstitial lung disease, neurological involvement, autoimmune thyroiditis, renal interstitial disease, anemia, leukopenia, hypergammaglobulinemia, and hypocomplementemia were more common in quadruple-seropositive patients with pSS than in quadruple-seronegative patients (*p* < 0.0001). The results of this study confirmed the strong impact of immunological markers on the pSS phenotype at the time of diagnosis. Immunological patterns play a central role in the phenotypic expression of the disease, even during the initial diagnostic phase, and can guide physicians in designing personalized treatment plans for patients with pSS.

## 1. Introduction

Sjögren’s syndrome (SS) is a systemic chronic autoimmune inflammatory disease that mainly affects the exocrine glands via lymphocytic infiltration, resulting in dryness of the eyes and mouth [1]. SS may occur alone (primary form-pSS) or overlap with another well-defined rheumatic disease (secondary form-sSS), indicating differences in pSS and sSS pathology. SS has a worldwide distribution and its phenotype may vary according to geolocation, race, and ethnicity. The clinical presentation of SS is heterogeneous, ranging from symptoms of sicca to systemic diseases [2]. SS is associated with a diversity of autoantibodies due to aberrant B cell activation, with anti-SSA/anti-Ro and anti-SSB/anti-La, RF, and ANA being the most commonly encountered [3]. Anti-Ro/SS-A antibody is present in 50–75% of SS patients, and in approximately half of them, anti-La/SS-B antibody was also detected [4]. The prevalence of ANA and RF was 50–89% and 38–61%, respectively, in the sera of pSS patients [4]. Autoantibodies might lead to the destruction of epithelial cells, causing gland hypofunction and the development of sicca symptoms, which may also lead to extraglandular manifestations [5]. Many organs other than the exocrine glands may be affected in patients with SS, including the skin and joints; the lungs, heart, and gastrointestinal tract, including the pancreas and liver; the kidneys, bladder, and gynecologic system; and both the peripheral nervous system and central nervous system [6]. Immunological markers provide prognostic information for in the diagnosis of the disease, prediction of the results, and extraglandular manifestations [7]. Previous studies have emphasized the association of anti-Ro/SSA antibodies with the development of extra-glandular manifestations such as cutaneous vasculitis, lung involvement, nephritis, and risk of lymphoma [8,9]. A different study indicated that RF may be associated with serological positivity for anti-Ro and anti-La, as well as with severe systemic disease, pulmonary involvement, renal disease, and corticosteroid use [10].

The relationship between anti-Ro/SS-A and anti-La/SS-B autoantibody positivity and clinical findings in patients with Sjögren’s syndrome was further investigated. However, there are few studies in the literature, especially on RF and ANA antibodies and clinical entities. Unfortunately, there have been no studies specifically on quadruple antibody positivity. The present study focused on identifying biomarkers and potential therapeutic targets of SS subsets that vary in their autoantibody profiles. To this end, we compared the clinical and immunological features of SS patients categorized into three serological subgroups: (1) positive for ANA, RF, anti-SSA/Ro and anti-SSB/La antibodies (quadruple-seropositive), (2) negative for ANA, RF, anti-SSA/Ro, and anti-SSB/La antibodies (quadruple-seronegative), and (3) positive for ANA and anti-SSA/Ro antibodies (double-seropositive).

In this study, we aimed to determine differences in clinical findings by comparing the quadruple antibody positivity group with patients with fewer antibodies and those with no antibodies.

## 2. Materials and Methods

### 2.1. Study Design, Setting, and Ethics

This comparative study was conducted in the Department of Rheumatology Eskişehir Cıty Hospital, Turkey, between December 2019 and May 2024. Ethical approval was obtained from the local ethics committee (decision date: 23 May 2024, decision no: 2024/30) and the study was performed in accordance with the ethical standards stated in the Declaration of Helsinki and its amendments. The method and purpose of the study were explained to all participants in detail, and written informed consent was obtained from each participant. Patients were invited for examination every 3 or 6 months, written informed consent was obtained from all participants at the time of application, and information was provided about the study.

### 2.2. Study Population

The study population included 402 consecutive patients with pSS (378 women and 24 men) who fulfilled the 2016 EULAR/ACR criteria [11]. The clinical characteristics of the patients were retrospectively obtained from their medical records. Laboratory information and pathological reports were obtained from the patient’s files in the hospital’s electronic database. As anti-Ro/SSA-positive patients fulfilled the EULAR criteria, minor salivary gland biopsy (MSGB) was not performed. However, MSGB was also performed in some SSA-positive patients who did not fulfill the EULAR criteria and whose sicca symptoms were not prominent. MSGB was performed in all patients with autoantibody positivity or negativity. The exclusion criteria were as follows: combined head and neck radiation, chronic hepatitis C or human immunodeficiency virus infections, prior lymphoproliferative disease, sarcoidosis, graft-versus-host disease, amyloidosis, IgG4-related disease, fibromyalgia and associated systemic autoimmune diseases, such as systemic lupus erythematosus, rheumatoid arthritis, mixed connective tissue disease, myopathies, and systemic sclerosis. In all patients, diseases that may cause sSS were excluded on the basis of serological tests and clinical findings. The levels of complement (C3 and C4), erythrocyte sedimentation rate (ESR), C-reactive protein (CRP), immunoglobulins (IgG, IgM, and IgA), anti-nuclear antibody (ANA), anti-dsDNA, rheumatoid factor (RF), anti-CCP antibody, and autoantibody-targeted extractable nuclear antigens (ENAs) were detected using the immune blot method, which identified seven different target autoantigens, including U1RNP/Sm, Sm, SS-A, Ro52, SS-B, Scl-70, and Jo-1 from the case records. Patients without insufficient data for SS diagnosis in the registry were excluded from the study. General procedural data and laboratory and clinical information at the onset of pSS, including the first presentation and systemic involvement, were retrospectively reviewed. Clinical data, such as age at diagnosis, disease duration, oral and ocular dryness, constitutional symptoms, and data on joint, pulmonary, kidney, vasculitis, skin, nervous, gastrointestinal tract, and endocrine involvement, were collected. Schirmer’s test was considered positive when less than 5 mm of paper was humid after 5 min. A minor salivary gland biopsy was performed on the lower lip through an incision. Focal lymphocytic sialadenitis in a minor salivary gland biopsy with one or more foci of lymphocytes per 4 mm2 (focus score ≥ 1) was accepted as the histopathological criterion [12].

The presence and titers of the ANAs were determined using an indirect immunofluorescence method with the Hep2 cell line as an antigen and an antibody titer test; the cutoff value for ANA was 1:80. The values used in our laboratory, anti-Ro/SSA, and anti-La/SSB were detected using enzyme immunoassay, while RF of IgM class (normal range < 30 IU/mL) and other autoantibodies were identified by routine laboratory tests. Patients with cytopenias were evaluated hematologically, and those with malignancies, leukemia, drugs, B12, folate, iron deficiency, or anemia of chronic disease were excluded. Leucopenia was defined as white blood cell count < 4.00 × 10^3^/mm^3^, neutropenia (neutrophil < 1.5 × 10^3^/mm^3^), anaemia (haemoglobin concentration < 12 g/dL), thrombocytopenia (platelet count < 150 × 10^3^/mm^3^).

In patients with petechiae and purpura, skin biopsies were performed after excluding drug reactions and xeroderma. To exclude other etiological causes in all patients with vasculitis, previous or current infections, antibiotics used, malignancy status, vaccinations received in the last six months, possible etiological factors, antineutrophil cytoplasmic antibodies (ANCA), immunodeficiency virus (HIV), hepatitis B virus (HBV), hepatitis C virus (HCV) syphilis serology, streptococcal antibodies, abdominal ultrasound examination, chest radiography, fecal occult blood test (FOBT), and skin biopsy were performed. Direct immunofluorescence (DIF) tests for IgA, IgG, IgM, and C3 were performed using skin biopsy. All patients with vasculitis were evaluated for medium- and large-vessel vasculitis, and systemic involvement. All patients were classified according to the 2012 Revised International Chapel Hill Consensus Conference (CHCC) Nomenclature of Vasculitides [13]. Cryoglobulinemic vasculitis was diagnosed when the serum cryoglobulins were positive with characteristic clinical features. Urticarial vasculitis is diagnosed when long-lasting (more than 24 h) indurated weals, which may be itchy, painful, tender, or accompanied by purpura, occur spontaneously or at sites of minor trauma.

Patients were grouped according to the following serological profiles: group 1, positive for ANA, RF, anti-SSA/Ro, and anti-SSB/La antibodies (quadruple-seropositive); group 2, negative for ANA, RF, anti-SSA/Ro, and anti-SSB/La antibodies (quadruple-seronegative); and group 3, positive for ANA and anti-SSA/Ro antibodies (double-seropositive).

### 2.3. Statistics

All statistical analysis was performed using Statistical Package for Social Sciences (SPSS) 23.0 software (IBM Corp., Armonk, NY, USA). Descriptive values are expressed as numbers (*n*) and percentages (%) for categorical values; they are expressed as the mean (standard deviation, SD) if normally distributed and as the median (interquartile range, IQR) if not normally distributed. For continuous variables, comparisons of median values were performed using the Mann–Whitney U-test. Student’s *t*-test was used as a parametric test. The Chi-square test and Fisher’s exact test were used for categorical variables. The statistical significance level was accepted as a *p*-value < 0.05 in all comparisons.

## 3. Results

### 3.1. Basic Characteristics of Total pSS Patients Included in the Present Study

The baseline characteristics of patients diagnosed with pSS are shown in Table 1. In total, 402 patients with pSS were retrospectively analyzed with a mean age of 54.3 (18–84) years and the patients were predominantly female 378 (94.0%). Among these patients, 78.8% (317/402) were seropositive (with at least one antibody positivity) and 85 (21.1%) were quadruple seronegative. Anti-SSA was present in 252 (62.6%) and anti-SSB in 75 (18.6%) patients. Other autoantibodies, including ANA and RF, were present in 284 (70.6%) and 96 (23.8%) of patients with pSS, respectively. The combined ANA and SSA positivity was 174 (43.2%). The number of quadruple-seropositive patients was 72 (18.6%). ANA staining pattern, with a granular pattern at 61%, nuclear at 20%, homogenous at 10%, centromere at 6%, and other staining patterns at 3%. MSGB was performed on a total of 232 patients and a focus score ≥ 1 was detected in 220 (94.8%) of them.

### 3.2. Comparison of Clinical Characteristics According to the Presence of Antibodies

Clinical characteristics and comparisons of seropositive, seronegative, and double-seropositive pSS are shown in Table 2 and Table 3. The age at diagnosis for quadruple-seropositive pSS was 42.4 ± 10.8, which was significantly younger than that of patients with double-seropositive and quadruple-seronegative pSS (*p* = 0.021, *p* = 0.112). When quadruple-seropositive pSS was compared with quadruple-seronegative, symptoms related to glandular dysfunction, such as xerostomia (97.2% vs. 94.1%, *p* = 0.112) and xerophthalmia (94.0% vs. 88.2%, *p* = 0.142), were more common in quadruple-seropositive pSS (Table 2). Similarly, when quadruple-seropositive pSS was compared with double seropositivity, xerostomia (97.2% vs. 92%, *p* = 0.242) and xerophthalmia (94.0% vs. 90.8%, *p* = 0.361) were more common in quadruple-seropositive pSS (Table 3). However, this similarity was not statistically significant between the double-seropositive and quadruple-seronegative groups (*p* = 0.345 and *p* = 0.526) (Table 4). In terms of organ involvement, salivary gland enlargement, arthralgias, arthritis, Raynaud’s phenomenon, lymphadenopathy, vasculitis-purpura, interstitial lung disease, neurological involvement, autoimmune thyroiditis, renal interstitial disease, anemia, leukopenia, hypergammaglobulinemia, and hypocomplementemia were more common in quadruple-seropositive than in quadruple-seronegative pSS (*p* < 0.0001) (Table 2). Except for salivary gland enlargement, arthralgia, and hypocomplementemia, other involvements were more frequent in quadruple-seropositive individuals than in those with double seropositivity (*p* < 0.0001) (Table 3). Similarly, when double seropositivity was compared with quadruple seronegativity, salivary gland enlargement, arthralgia, arthritis, lymphadenopathy, and interstitial lung disease were more common in double seropositivity (*p* < 0.0001) (Table 4). Interstitial lung disease was observed in 98.2% (55/56) of seropositive (at least one antibody positive) patients, while it was 1.7% (1/56) in seronegative patients (all antibodies negative) (*p* < 0.0001). NSIP accounted for 89.2% of the ILD patterns, and almost all of them were observed in seropositive patients. Neurological involvement was observed in 95.0% (19/20) of seropositive patients, while it was 5.0% (1/20) of seronegative patients (*p* < 0.0001). Autoimmune thyroiditis was present in 86 patients (21.3%); 74.4% of these patients were ANA positive and ANA staining: granular pattern, 84%; homogenous at 10%; and nuclear, 6%. Of the autoimmune thyroiditis patients, 79 (91.8%) had Hashimoto’s disease, five had Graves’ disease, and two had subacute thyroiditis. Moreover, autoimmune thyroiditis was observed in 83.7% (72/86) of seropositive patients, while it was 9.3% (8/86) of seronegative patients (*p* < 0.0001). Cryoglobulinemia, Raynaud’s phenomenon, vasculitis, and renal interstitial disease were not observed in any of the quadruple-seronegative patients. In our patients who were diagnosed with Sjögren’s syndrome and had symptoms such as cough and dyspnea, chest computed tomography (CT) was performed. Patients without any of these symptoms underwent lung radiography. CT was performed for patients with interstitial lung findings.

The laboratory findings of quadruple-seropositive and quadruple-seronegative pSS patients were also compared (Table 2). ESR and CRP levels were lower in the seronegative pSS group (*p* = 0.125 vs. *p* = 0.362). Peripheral blood parameters including platelet count (*p* = 0.001), hemoglobin (*p* < 0.0001), leukocyte count (*p* < 0.0001), and lymphopenia (*p* < 0.0001) levels were lower in quadruple-seropositive pSS. The level of hypergammaglobulinemia was higher in individuals with quadruple seropositivity than in those with quadruple seronegativity (*p* < 0.0001). No significant differences were observed between quadruple-seronegative and quadruple-seropositive pSS in other laboratory parameters, such as creatinine level, and variables related to liver, renal, and thyroid function. Twelve patients had malignancies, including six patients with breast cancer, one with thyroid follicular cancer, two with colon cancer, one with ovarian cancer, one with melanoma, and one with diffuse large B-cell lymphoma. Malignancy was also observed in both seropositive and seronegative pSS patients and was not statistically significant. No correlation was observed between the autoantibody positivity and malignancy. However, patients with lymphoma are positive for ANA, anti-SSA, and anti-SSB antibodies. Hydroxychloroquine could not be administered to eight patients due to contraindications and to seven patients due to side effects, but it was administered to all other patients. There were no significant differences in the frequency of corticosteroid, methotrexate, hydroxychloroquine sulfate, or azathioprine treatment among the three groups, although the use of pilocarpine hydrochloride was significantly higher in the seropositive group.

Vasculitis was also observed in 20 patients. Of these, 12 had a previous diagnosis of pSS, and eight were diagnosed with pSS during the investigation of vasculitis etiology. After excluding other causes of vasculitis (such as large, medium, and small vessel vasculitis), it was accepted as Sjögren syndrome-associated small vessel vasculitis (SS-SVV) from the vasculitis associated with the systemic disease group. Of these, 15 had leukocytoclastic vasculitis (LCV), three had cryoglobulinemic vasculitis (CV), and two had urticarial vasculitis. There were 10 quadruple-seropositive pSS patients, five double-seropositive, two isolated RF positive, and three quadruple-seronegative patients with vasculitis. Patients with cutaneous vasculitis had a higher prevalence of ANA positivity (75% vs. 70.4%, *p* = 0.012), RF (65% vs. 22.5%, *p* < 0.0001), anti-Ro/SS-A antibodies (70% vs. 62.3%, *p* = 0.014), anti-La/SS-B antibodies (55% vs. 16%, *p* < 0.0001) compared with pSS patients without vasculitis. All the patients with LCV received oral corticosteroids and hydroxychloroquine. Two patients received corticosteroid doses > 30 mg/day, whereas the others went into remission with lower doses of treatment. Eight patients received immunosuppressive agents (one cyclophosphamide and seven azathioprine). Two patients with urticarial vasculitis were treated with azathioprine. Oral corticosteroids and hydroxychloroquine were administered to three patients with CV, and cyclophosphamide then azathioprine to one patient, azathioprine to one patient, and methotrexate was administered to one patient with predominant arthritis. The average follow-up period for patients with CV was only 14 months, and the patients were periodically examined for lymphoma. Fourteen patients had a single episode of cutaneous vasculitis, and the remaining six had relapsing vasculitis. All our patients with vasculitis survived.

## 4. Discussion

The clinical presentation of pSS is highly heterogeneous [14]. Patients may present with varying clinical manifestations, ranging from sicca symptoms to systemic diseases and lymphoma [2,15]. Anti-SSA and anti-SSB are the most commonly used immunologic biomarkers for the diagnosis of pSS; however, a proportion of patients with pSS are negative for both autoantibodies, and patients have varying levels of RF and ANA antibodies. These autoantibodies cause epithelial cell damage, leading to the development of symptoms [5]. A link between pathological salivary gland ultrasound (SGU) findings and positive autoimmunity was established, and studies have shown that SGU is a strong predictor of positive SGU findings in patients with sicca symptoms [16]. In a similar study conducted according to the number of autoantibodies, pathological SGU findings were observed more frequently in patients with quadruple-seropositive antibodies. The quadruple-seropositive group had the highest frequency of pathological SGU findings (78.1%). Most patients in the partially seropositive (with at least one of the four autoantibodies) group (60.7%) and all seronegative patients had normal SGU findings [17]. These studies have provided information on antibody-associated organ damage. The present study investigated the clinical heterogeneity of Turkish patients with pSS and found that patients with quadruple; double antibodies were significantly different from seronegative pSS patients in several respects. The age at diagnosis of quadruple-seropositive pSS patients was significantly younger than that of double-seropositive and quadruple-seronegative patients. The interval between the onset of sicca symptoms and diagnosis was the shortest in quadruple-seropositive patients, averaging 23 months, whereas it was the longest in quadruple-seronegative patients, averaging 60 months (*p* < 0.0001). This shows that the presence of autoantibodies in patients with pSS aids early diagnosis. In addition, those with antibody positivity are diagnosed earlier because of their higher disease activity and higher systemic involvement. We found that patients who tested positive for autoantibodies were referred to specialists for autoimmune diseases and were diagnosed earlier. The first interesting finding of our study was the prevalence of patients with quadruple-seropositive reaching almost 17.9% and quadruple-seronegative 21.4% of the total SS population.

When comparing patients with quadruple-seropositive and double-seropositive pSS, the double-positive group was older. Clinical findings such as sicca symptoms and arthralgia were predominant; however, systemic involvement such as salivary gland enlargement, arthritis, Raynaud’s phenomenon, lymphadenopathy, vasculitis, interstitial lung disease, neurological involvement, autoimmune thyroiditis, primary biliary cholangitis, cytopenia, and hypergammaglobulinemia were less common. The difference between quadruple-seropositive and double-seropositive is RF and anti-SSB positive, extraglandular involvement is more in quadruple-seropositive and we think that this is more related to anti-SSB positivity (Table 2 and Table 3). We believe that high disease activity and systemic organ involvement are associated with anti-SSB/La. Previous studies have shown that anti-SSB/La antibodies trigger immunoreactivity, increase systemic antibody production, and cause tissue damage by inducing inflammatory reactions [18,19,20,21]. Arthritis and arthralgia were more common in the quadruple-seropositive group than in the other groups, and we believe that RF positivity was a contributing factor. Supporting this, a previous study found that the frequency of articular manifestations was higher in RF-positive patients with pSS than in RF-negative patients [4].

In general, the presence of autoantibodies correlates with a younger age of onset, female predominance, increased risk of organ involvement, and the presence of other antibodies [22]. In our study, comparing patients with quadruple-seropositive and quadruple-seronegative pSS, the seronegative group was older, and clinical findings and systemic involvement were significantly less common (Table 2). This finding is important because it shows that seropositive antibodies are more likely to develop severe diseases. Autoantibody status is the main factor driving the phenotypic expression of pSS and may help identify subgroups of patients with poor prognosis [3].

Cutaneous vasculitis is observed in approximately 4–10 % of patients with pSS [23,24,25,26] One possible approach for classifying SS-associated small vessel vasculitis (SVV) is to distinguish between cryoglobulinemic vasculitis, urticarial vasculitis, and non-cryoglobulinemic, non-urticarial leukocytoclastic vasculitis [27]. In the same study, CV was diagnosed in 14 (27%) of the 52 SS patients with cutaneous vasculitis [27]. The prevalence of cryoglobulins in pSS is 9–15%, the most common type being type III mixed cryoglobulinemia [28]. In our study, cryoglobulinemia was positive in three patients (0.7%). Three patients had cutaneous vasculitis with hypocomplementemia and were in the quadruple-seropositive group. In our study, there were fewer cases of cryoglobulinemia compared with other studies. The reason for this is that we only studied patients with vasculitis, neurological involvement, and RF positivity together with hypocomplementemia. We observed that the lesions were more generalized and accompanied by necrosis in CV than in other patients with vasculitis. In the same study, LCV was diagnosed in the remaining 26 (50%) patients and urticarial vasculitis was diagnosed in 11 (21%) of the 52 pSS patients with cutaneous vasculitis [27]. In our study, LCV was observed in 15 (75%) and urticarial vasculitis in two (10%). We can associate these differences with the increase in antibody positivity; because the number of autoantibodies was higher in our study. We observed that the duration of remission in patients with hypocomplementemia was short, and relapses were frequent. Additionally, higher doses of corticosteroids were administered to these patients. Based on these data, more aggressive and effective immunosuppressive treatments may need to be initiated in patients with hypocomplementemia. The development of cutaneous vasculitis may have significant prognostic implications, as such patients are more likely to develop other extraglandular manifestations, including lymphoma, and die from disease-related complications than those without vasculitis [27,29,30]. Raynaud’s phenomenon, vasculitis, renal interstitial disease, and cryoglobulinemia were not observed in the quadruple-seronegative patients. These findings suggest that autoantibody-related and circulating immune complexes play a major role in organ damage and may be more frequent in the seropositive group. However, the clinical expression of the disease should not be interpreted solely based on autoantibodies, and the counter-immunoregulation and genetic features should also be considered; therefore, not all seropositive patients are expected to develop a worse clinical phenotype.

Our study has several limitations. This study was conducted at a single center in Turkey, with a retrospective design and the sample size was relatively small, particularly for pSS-associated small vessel vasculitis. The relationship between disease activity and systemic involvement could not be evaluated because of the retrospective nature of the study. Cryoglobulinemia was studied only in vasculitis patients with neurological involvement and in patients with RF positivity and hypocomplementemia. As it has not been studied in other patients, we encountered it less frequently than in previous studies. We could not make a histological comparison between the three groups because they were evaluated by different pathologists, and most had focus scores of >1 or an aggregate number of >1. The follow-up period was 18 months; because of this short follow-up period, we could not observe any long-term complications. We were unable to perform bone marrow biopsies of patients with cytopenia because they did not provide their consent. ESSPRI or ESSDAI scoring could not be performed because our study was retrospective and some data were missing.

## 5. Conclusions

Extraglandular manifestations are more prevalent in quadruple-seropositive patients than in double-seropositive and quadruple-seronegative patients with pSS. We believe that antibody positivity is a significant factor, particularly in circulating immune complexes and antibody-related organ damage such as vasculitis, Raynaud’s phenomenon, and cryoglobulinemia. The results of this study confirmed the strong influence of immunological markers on the phenotype of pSS at diagnosis. Immunological patterns play a crucial role in the phenotypic expression of the disease, even during the initial diagnostic phase. Consequently, they can guide physicians in designing personalized treatment plans for patients with pSS.

## Figures and Tables

**Table 1:** Baseline characteristics of 402 patients with primary Sjögren’s syndrome.

Demographic Features	Patients (%)
Age, median (range), years	54.3 (18–84)
Gender (female/male)	378/24
Age of diagnosis (mean ± SD)	48.3 ± 14.8
Disease duration, median (range), months	18 (1–54)
**Clinical features**	
Xerostomia	372 (92.5%)
Xerophtalmia	360 (89.5%)
**Schirmer**	
≤5 mm	240 (59.7%)
5–10 mm	100 (24.8%)
≥10 mm	62 (15.4%)
Focus score ≥ 1	220 (94.8%)
Salivary gland enlargement	52 (12.9%)
Arthralgias	244 (60.6%)
Arthritis	64 (15.9%)
Raynaud’s phenomenon	16 (3.9%)
Lymphadenopathy	36 (8.9%)
Vasculitis-purpura	20 (4.9%)
Interstitial lung disease	56 (13.9%)
NSIP	50/56 (89.2%)
OP	1/56 (1.7%)
UIP	5/56 (8.9%)
Neurological involvement	20 (4.9%)
Central nervous system involvement	12/20 (60%)
Peripheral nervous involvement	8/20 (40%)
Autoimmune thyroiditis	86 (21.3%)
Primary biliary cholangitis	10 (2.4%)
Renal interstitial disease	2 (0.4%)
Malignancy	12 (2.9%)
**Laboratory features**	
ANA positivity	284 (70.6%)
RF positivity	96 (23.8%)
SSA positivity	252 (62.6%)
SSB positivity	75 (18.6%)
Quadruple-seropositive	72 (17.9%)
Quadruple-seronegative	85 (21.4%)
ANA and SSA positivity	174 (43.2%)
ESR (mm/h) (mean ± SD)	35.6 ± 20.7
CRP (mg/L) (mean ± SD)	12.3 ± 3.2
Anemia	76 (18.9%)
Leukopenia	42 (10.4%)
Lymphopenia	56 (13.9%)
Thrombocytopenia	22 (5.4%)
Cryoglobulinaemia	3 (0.7%)
Hypocomplementemia	20 (4.9%)
Hypergammaglobulinemia	48 (11.9%)
**Treatments**	
Corticosteroids	106 (26.3%)
Hydroxychloroquine	387 (96.2%)
Methotrexate	28 (6.9%)
Azathioprine	18 (4.4%)
Cyclophosphamide	4 (0.9%)
Pilocarpine	42 (10.4%)

NSIP: non-specific interstitial pneumonia, OP: organizing pneumonia, UIP: usual interstitial pneumonia.

**Table 2:** Comparison of patients with quadruple seropositivity and quadruple seronegativity.

Feature/Clinical Manifestation	Quadruple-Seropositive *n*: 72, %	Quadruple-Seronegative *n*: 85, %	*p*-Value
Age, median (range), years	47.9 (18–72)	59.2 (20–82)	0.031
Female	70 (97.2%)	78 (91.7%)	0.125
Age of diagnosis (mean ± SD)	42.4 ± 10.8	50.3 ± 13.2	0.112
Xerostomia	70 (97.2%)	80 (94.1%)	0.328
Xerophtalmia	68 (94.0%)	75 (88.2%)	0.142
Salivary gland enlargement	20 (27.7%)	3 (3.5%)	<0.0001
Arthralgias	60 (83.3%)	22 (25.8%)	<0.0001
Arthritis	32 (44.4%)	2 (2.3%)	<0.0001
Raynaud’s phenomenon	11 (15.2%)	0	<0.0001
Lymphadenopathy	21 (29.1%)	2 (2.3%)	<0.0001
Interstitial lung disease	28 (38.8%)	1 (1.1%)	<0.0001
Vasculitis	10 (13.8%)	3 (3.5%)	<0.0001
Neurological involvement	14 (19.4%)	1 (1.1%)	<0.0001
Autoimmune thyroiditis	42 (58.3%)	8 (9.4%)	<0.0001
Primary biliary cholangitis	6 (8.3%)	1 (1.1%)	0.023
Renal interstitial disease	2 (2.7%)	0	0.587
Malignancy	5 (6.9%)	2 (2.3%)	0.016
Cryoglobulinaemia	3 (4.1%)	0	0.348
ESR (mm/h) (mean ± SD)	43.1 ± 14.3	32.6 ± 16.8	0.125
CRP (mg/L) (mean ± SD)	10.2 ± 4.4	6.3 ± 8.7	0.362
Anemia	32 (44.4%)	10 (11.7%)	<0.0001
Leukopenia	20 (27.7%)	4 (4.7%)	<0.0001
Lymphopenia	24 (33.3%)	12 (14.1%)	0.001
Thrombocytopenia	10 (13.8%)	2 (2.3%)	0.001
Hypergammaglobulinemia	34 (47.2%)	4 (4.7%)	<0.0001
Hypocomplementemia	5 (6.9%)	8 (9.4%)	0.352

**Table 3:** Comparison of quadruple-seropositive and double-seropositive patients.

Feature/Clinical Manifestation	Quadruple-Seropositive *n*: 72, %	Double-Seropositive *n*: 174, %	*p*-Value
Age, median (range), years	47.9 (18–72)	54.2 (24–84)	0.091
Female	70 (97.2%)	164 (94.2%)	0.426
Age of diagnosis (mean ± SD)	42.4 ± 10.8	52.3 ± 14.2	0.021
Xerostomia	70 (97.2%)	161 (92.5%)	0.242
Xerophtalmia	68 (94.0%)	158 (90.8%)	0.361
Salivary gland enlargement	20 (27.7%)	22 (12.6%)	0.113
Arthralgias	60 (83.3%)	138 (79.3%)	0.516
Arthritis	32 (44.4%)	16 (9.1%)	<0.0001
Raynaud’s phenomenon	11 (15.2%)	5 (2.8%)	<0.0001
Lymphadenopathy	21 (29.1%)	12 (6.8%)	<0.0001
Interstitial lung disease	28 (38.8%)	22 (12.6%)	<0.0001
Vasculitis	10 (13.8%)	5 (2.8%)	<0.0001
Neurological involvement	14 (19.4%)	5 (2.8%)	<0.0001
Autoimmune thyroiditis	42 (58.3%)	30 (17.2%)	<0.0001
Primary biliary cholangitis	6 (8.3%)	4 (2.2%)	0.001
Renal interstitial disease	2 (2.7%)	0	0.657
Malignancy	5 (6.9%)	3 (1.7%)	0.325
Cryoglobulinaemia	3 (4.1%)	0	0.388
ESR (mm/h) (mean ± SD)	43.1 ± 14.3	36.4 ± 10.8	0.125
CRP (mg/L) (mean ± SD)	10.2 ± 4.4	7.6 ± 5.7	0.362
Anemia	32 (44.4%)	24 (13.7%)	<0.0001
Leukopenia	20 (27.7%)	15 (8.6%)	<0.0001
Lymphopenia	24 (33.3%)	20 (11.4%)	<0.0001
Thrombocytopenia	10 (13.8%)	8 (4.5%)	<0.0001
Hypergammaglobulinemia	34 (47.2%)	14 (8.0%)	<0.0001
Hypocomplementemia	5 (6.9%)	7 (4.0%)	0.456

**Table 4:** Comparison of double-seropositive patients with quadruple-seronegative patients.

Feature/Clinical Manifestation	Double- Seropositive *n*: 174, %	Quadruple-Seronegative *n*: 85, %	*p*-Value
Age, median (range), years	54.2 (24–84)	59.2 (20–82)	0.162
Female	164 (94.2%)	78 (91.7%)	0.386
Age of diagnosis (mean ± SD)	52.3 ± 14.2	50.3 ± 13.2	0.461
Xerostomia	161 (92.5%)	80 (94.1%)	0.345
Xerophtalmia	158 (90.8%)	75 (88.2%)	0.526
Salivary gland enlargement	22 (12.6%)	3 (3.5%)	<0.0001
Arthralgias	138 (79.3%)	22 (25.8%)	<0.0001
Arthritis	16 (9.1%)	2 (2.3%)	<0.0001
Raynaud’s phenomenon	5 (2.8%)	0	0.623
Lymphadenopathy	12 (6.8%)	2 (2.3%)	<0.0001
Interstitial lung disease	22 (12.6%)	1 (1.1%)	<0.0001
Vasculitis	5 (2.8%)	3 (3.5%)	0.568
Neurological involvement	5 (2.8%)	1 (1.1%)	0.084
Autoimmune thyroiditis	30 (17.2%)	8 (9.4%)	0.032
Primary biliary cholangitis	4 (2.2%)	1 (1.1%)	0.021
Renal interstitial disease	0	0	null
Malignancy	3 (1.7%)	2 (2.3%)	0.612
Cryoglobulinaemia	0	0	null
ESR (mm/h) (mean ± SD)	36.4 ± 10.8	32.6 ± 16.8	0.421
CRP (mg/L) (mean ± SD)	7.6 ± 5.7	6.3 ± 8.7	0.512
Anemia	24 (13.7%)	10 (11.7%)	0.536
Leukopenia	15 (8.6%)	4 (4.7%)	0.004
Lymphopenia	20 (11.4%)	12 (14.1%)	0.426
Thrombocytopenia	8 (4.5%)	2 (2.3%)	0.002
Hypergammaglobulinemia	14 (8.0%)	4 (4.7%)	0.004
Hypocomplementemia	7 (4.0%)	8 (9.4%)	0.001

## Data Availability

The corresponding datasets of this study are available from the corresponding author upon reasonable request.
